# Soehendra stent retriever as a useful delivery device of drainage stent for passing an impacted cystic duct stone in a patient with acute cholecystitis

**DOI:** 10.1002/deo2.78

**Published:** 2021-12-24

**Authors:** Tesshin Ban, Yoshimasa Kubota, Takuya Takahama, Tomoaki Ando, Takashi Joh

**Affiliations:** ^1^ Department of Gastroenterology and Hepatology Gamagori Municipal Hospital Aichi Japan

**Keywords:** acute cholecystitis, endoscopic transpapillary gallbladder drainage, Soehendra stent retriever

## Abstract

Endoscopic transpapillary gallbladder drainage is an alternative procedure for patients with acute cholecystitis. However, this procedure is technically challenging because the drainage stent is sometimes obstructed by an impacted cystic duct stone, even if the guidewire is advanced into the gallbladder. In this report, the front end of a standard endoscopic retrograde cholangiopancreatography catheter was cut to an appropriate length as a drainage stent for transpapillary gallbladder drainage. However, this modified stent became stuck because of an impacted cystic duct stone. The Soehendra stent retriever was used as a stent delivery device in this setting. A Soehendra stent retriever with clockwise rotation was coupled with the drainage stent. Integrated devices provide a stent tip for pushability and torqueability. The stuck drainage stent at the impacted cystic duct stone resumed advancement into the gallbladder. After stent indwelling, decoupling was easy under counterclockwise rotation of the Soehendra stent retriever.

## INTRODUCTION

In patients with moderate/grade II acute cholecystitis (AC), urgent laparoscopic cholecystectomy (Lap‐C) is recommended after onset based on the patient's performance status.^1^ However, in the current situation, gastroenterologists have no choice but to consider biliary drainage before surgery based on multiple factors. Percutaneous transhepatic gallbladder drainage (PTGBD) is recommended.^1^ Additionally, endoscopic transpapillary GBD (ETGBD)^2^ and endoscopic ultrasonography‐guided GBD (EUS‐GBD)^3^ are now technically available as alternatives to biliary drainage. However, PTGBD impairs patients’ quality of life due to a worrisome external biliary drainage tube. Recently, ETGBD was used for temporal drainage prior to the Lap‐C.^4^ However, ETGBD has some drawbacks, including a relatively low technical success rate (82.6%) and a relatively high incidence of adverse events (8.83%).^5^ Technical success depends on the presence of cystic duct stones, dilation of the common bile duct, and cystic duct direction.^6^

The Soehendra stent retriever (SSR; COOK Medical, Bloomington, USA) was originally developed for transpapillary biliary plastic stent exchange.^7^ In recent reports, SSR has been exclusively used as a dilation device or an access route creator.^8^, ^9^

In this study, we report the first case of AC in which the ETGBD stent was advanced using the SSR as an indweller to pass the impacted cystic duct stone.

## CASE REPORT

**FIGURE 1:**
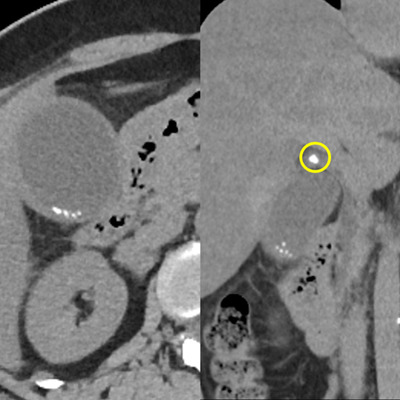
Abdominal computed tomography 4 days after admission. Swollen gallbladder with gallstones and wall thickness. The yellow circle in the coronal section indicates an impacted cystic duct stone

A 56‐year‐old female office worker was admitted to our emergency unit with epigastralgia. She presented with normal body temperature (36.6°C), high blood pressure (143/72 mmHg), normocardia (73 beats/min), O_2_ saturation rate of 99% at room air, and a Glasgow coma scale score of 15. The patient had an unremarkable previous history. Three months prior to this admission, she presented with cholelithiasis and choledocholithiasis. She underwent endoscopic lithotripsy following endoscopic sphincterotomy and was scheduled to undergo Lap‐C in 2 months at another institution. Blood test results upon admission indicated signs of infection with a white blood cell count of 7400 cells/μl and a C‐reactive protein of 19.4 mg/dl. Mildly elevated liver transaminase levels without jaundice were as follows: aspartate aminotransferase, 45 IU/L; alanine aminotransferase, 53 IU/L; total bilirubin, 1.0 mg/dl; and direct bilirubin, 0.4 mg/dl. Renal function was within the normal range as follows: blood urea nitrogen, 8.3 mg/dl, and creatinine, 0.67 mg/dl. Platelet count was 28.3 × 10^4^ /μl and prothrombin time‐international normalized ratio was 1.02, both within the normal range. Additionally, computed tomography (CT) upon admission revealed a swollen gallbladder with gallstones. In line with the Tokyo Guidelines 2018,^1^ a diagnosis of mild/grade I AC was made. The patient underwent intravascular fluid replacement and was administered antibiotics under fasting conditions. However, her abdominal pain deteriorated to rebound tenderness. On the second CT performed 4 days after the admission, the gallbladder was still swollen and edematous, and an impacted stone was observed in the cystic duct (Figure 1). After 72 h of persistent symptoms, the severity worsened to moderate/grade II AC.^1^ She refused to undergo urgent Lap‐C or PTGBD but consented to ETGBD for personal reasons.

**FIGURE 2:**
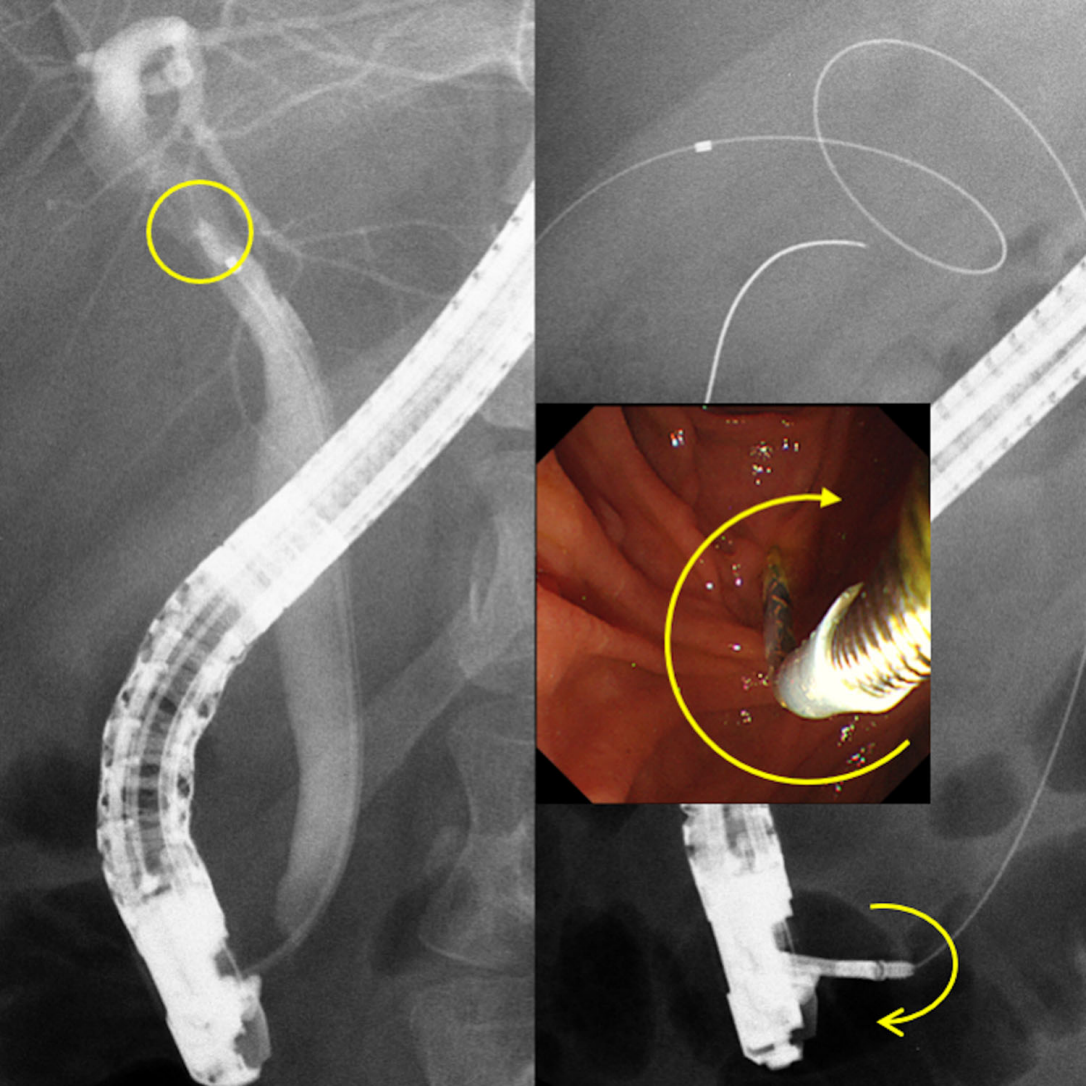
Cholangiography of negotiation at impacted cystic duct stone in endoscopic transpapillary gallbladder drainage. Left; crab's claw‐like impacted cystic duct stone with a yellow circle. Right: clockwise‐rotated Soehendra stent retriever (SSR) (yellow arrow) enables disconnected cholangiography catheter to pass the impacted cystic duct stone.

Cholangiography revealed a crab's claw‐like impacted stone at the first inflection point of the cystic duct (Figure 2). A standard endoscopic retrograde cholangiopancreatography (ERCP) catheter (MTW tapered ERCP catheter 0120211; MTW Endoskopie, Wesel, Germany) loaded with a 0.025‐inch hydrophilic‐tipped guidewire (Visiglide 2 guidewire; Olympus, Tokyo, Japan) was advanced into the gallbladder. Next, 90 ml of the infected bile was aspirated, and the gall bladder was irrigated with saline. However, a 6‐Fr nasobiliary drainage tube (Flexima; Baston Scientific, Marlborough, USA) became stuck because of the impacted stone.

**FIGURE 3:**
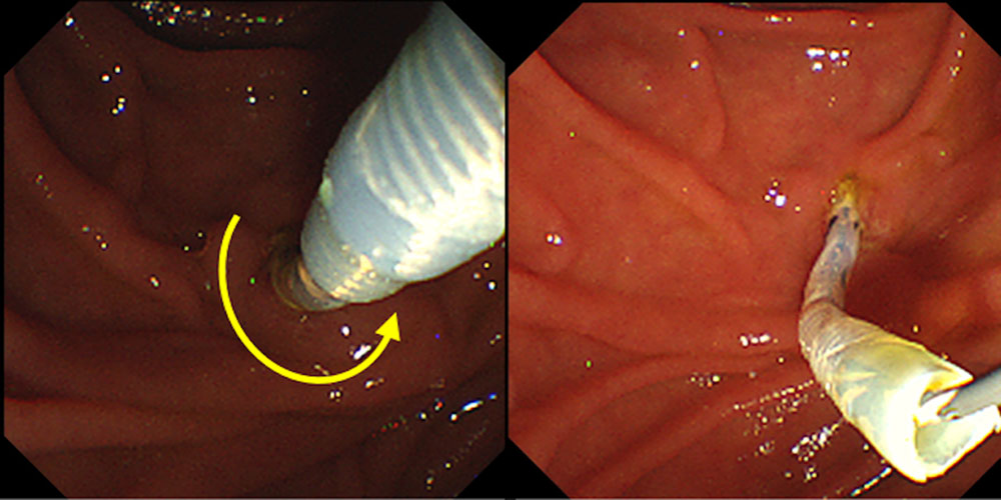
Release of the disconnected catheter from Soehendra stent retriever. Counterclockwise rotation of SSR (yellow arrow) enables catheter release. SSR: Soehendra stent retriever

An ERCP catheter that had previously passed through the impacted stone was cut 14 cm from the tip and used as an internal drainage stent. However, it also became lodged. Originally, a pusher catheter was changed to SSR (SSR‐7; COOK Medical) to remove the stuck drainage stent. The SSR handle was turned clockwise until it was screwed to the end of the drainage stent. The stent tip was advanced into the gallbladder by continuing to push and twist the SSR clockwise (Figure 2). Subsequently, the SSR was successfully detached by counterclockwise rotation (Figure 3). The total procedure time was 64 min, and the indwelling of this modified ERCP catheter took 10 min. No adverse events occurred during the procedure.

**FIGURE 4:**
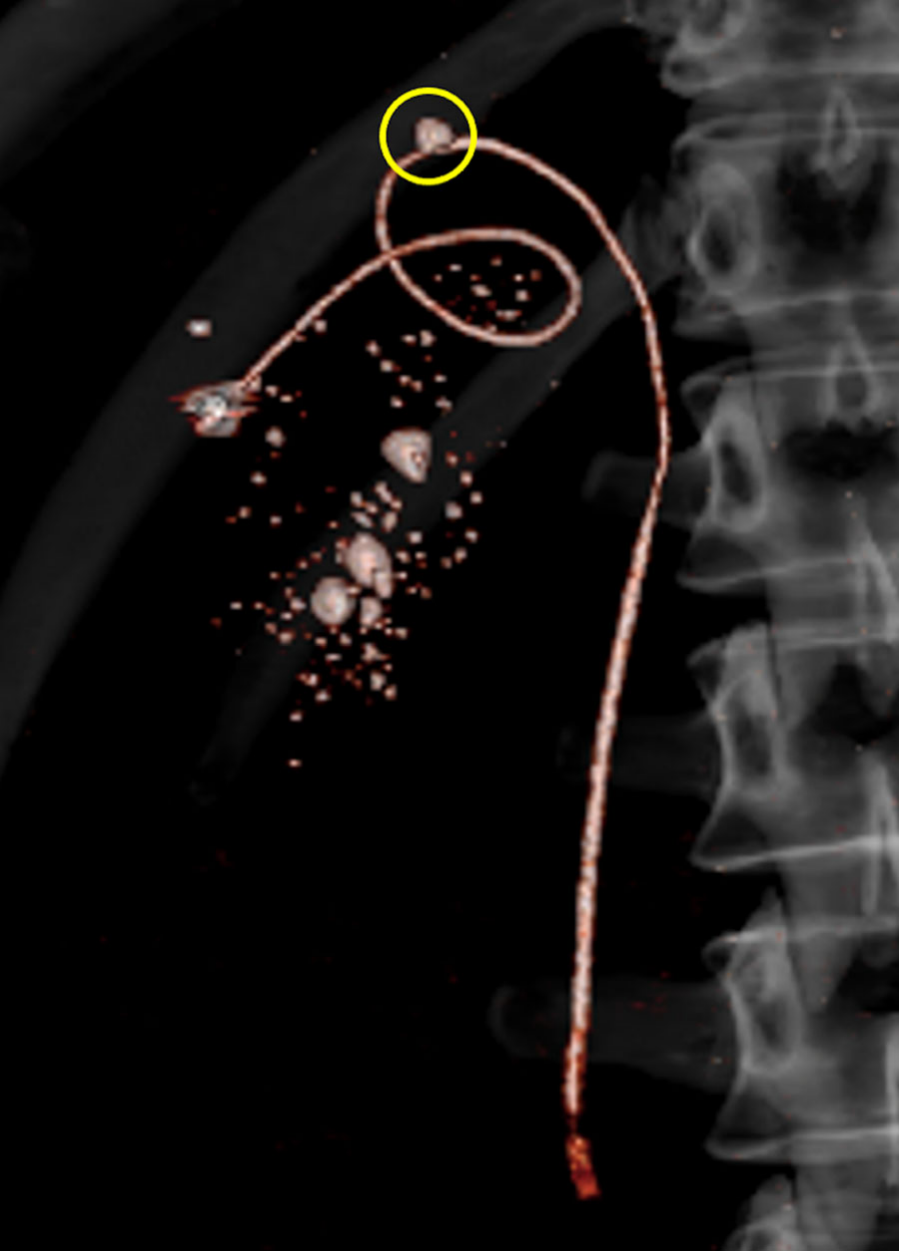
3D computed tomography imaging after the procedure. Disconnected cholangiography catheter passes the impacted cystic duct stone (yellow circle)

Abdominal CT performed the following day confirmed that the front end of the modified ERCP catheter was left in place as an ETGBD stent (Figure 4). The patient was discharged 3 days after the procedure, with an uneventful course up to the scheduled Lap‐C 40 days after discharge. The drainage stent was removed immediately before Lap‐C.

## DISCUSSION

Herein, we report the first case of AC drained using the front end of an ERCP catheter that was screwed into the gallbladder by SSR.

In line with the Tokyo Guidelines 2018,^1^ urgent Lap‐C or PTGBD is recommended for moderate/grade II AC. However, gastroenterologists avoid these procedures for several reasons. In this setting, ETGBD was used as an alternative. ETGBD includes endoscopic nasogallbladder drainage (ENGBD) and gallbladder stenting, with no significant difference in technical success, clinical success, or adverse event rates.^10^ However, in the present case, the 6‐Fr ENGBD tube could not pass through the gallbladder due to the impacted cystic duct stone. At this point, we abandoned the 5‐Fr ENGBD because of flaccidity and diameter of 1.67 mm.

For the ETGBD, we handcrafted a gallbladder drainage stent using a 1.6‐mm tip‐ERCP catheter modified to be cut 14 cm in length and used SSR as a stent delivery device. There are three steps to this procedure. The first is a coupling of the SSR and drainage stent in the scope channel; the second is stent indwelling into the gallbladder, and the third is decoupling of both. After coupling, insertion of the SSR under clockwise twisting conferred this drainage stent with both pushability and torqueability. After stent indwelling, decoupling was easy with counterclockwise rotation of the SSR. If this drainage stent could not be advanced, the SSR could be used to easily remove the drainage stent. Generally, the SSR and plastic stents are firmly connected. The mechanism and tips to release this modified stent are as follows: if both devices are in a straight shape, this stent would just rotate through the counterclockwise rotation of the SSR. However, as shown in Figures 2 and 4, the advanced part of the drainage stent is curved and stays away from the axis of the vector of removal. Through the counterclockwise rotation of the SSR, in addition to the axis rotation, the advanced part of the stent loop turns around the axis. However, the gallbladder and cystic duct counteracted the stent loop. The same is true for the connection of both devices. In addition, locking of the drainage stent using an elevator of the duodenoscope would assist device release.

Recent reports and studies exclusively used SSR as a dilator or access route creator.^8^, ^9^ In the present study, SSR was found to be a useful delivery device for ETGBD.

However, we were concerned about how long this small‐diameter ETGBD drainage stent would continue to function. This modified ERCP catheter was small in diameter and had no side holes. In a recent meta‐analysis, the rate of cholecystitis recurrence following ETGBD was reported to be 1.48% in inoperable patients with AC.^5^ This rescue technique warrants further study to investigate the technical success rate, clinical success rate, and adverse events.

In conclusion, SSR was a useful delivery device for a stuck drainage stent with an impacted cystic duct stone in a patient with AC.

## CONFLICT OF INTEREST

The authors declare no conflict of interest.

## FUNDING INFORMATION

This research did not receive any specific grant from funding agencies in the public, commercial, or not‐for‐profit sectors.

## ETHICS STATEMENT

The patient provided written informed consent for publication of details of the case, and the Gamagori Municipal Hospital Institutional Review Board granted permission to review the medical records. This study was conducted in accordance with the principles of the Declaration of Helsinki.
